# Droplet Deposition Distribution Prediction Method for a Six-Rotor Plant Protection UAV Based on Inverse Distance Weighting

**DOI:** 10.3390/s22197425

**Published:** 2022-09-29

**Authors:** Bin Wang, Yan Zhang, Chunshan Wang, Guifa Teng

**Affiliations:** 1 School of Information Science and Technology, Hebei Agricultural University, Baoding 071001, China; 2 School of Sciences, Hebei Agricultural University, Baoding 071001, China; 3 Hebei Key Laboratory of Agricultural Big Data, Baoding 071001, China; 4 Beijing Research Center for Information Technology in Agriculture, Beijing 100097, China; 5 National Engineering Research Center for Information Technology in Agriculture, Beijing 100097, China

**Keywords:** CFD, plant protection UAV, discrete phase, droplet deposition distribution, IDW

## Abstract

The aim of this work is to establish a method for real-time calculating droplet deposition distribution of a six-rotor plant protection unmanned aerial vehicle (UAV). The numerical simulation of the airflow field was carried out using computational fluid dynamics (CFD). The airflow field distribution was obtained under seven flight speeds, six flight heights, and seven crosswind speeds. The relative error verified the accuracy of the numerical model within 12% between the spatial point wind speed test and the simulated value. The numerical simulation results showed that with the improvement of the UAV flight speed and the crosswind, the relative airflow produces a vortex in the downwash wind field below the UAV and reduces the stability of the downwash wind field. The discrete droplet phase was introduced in the flow field. The ground regions were divided using a small grid of 0.5 m × 0.5 m, and statistical calculations of droplet deposition rates within each grid yielded the distribution of droplets under 294 different parameter combinations. The statistical results show that the relative airflow and crosswind caused droplet convolution, and droplet drift was increased. In the actual operation of the UAV, the flight speed should be well controlled under the condition of low environmental wind to reduce the droplet drift rate and improve the utilization rate of pesticides. Based on the distribution under 294 different parameter combinations, one droplet deposition prediction method was established using inverse distance weighting (IDW). The proposed method lays a foundation for the cumulative calculation of droplet deposition distribution during continuous operation of plant protection UAV. It provides a basis for objectively evaluating the operational quality of plant protection UAVs and optimizing the setting of operation parameters.

## 1. Introduction

**Figure 1:**
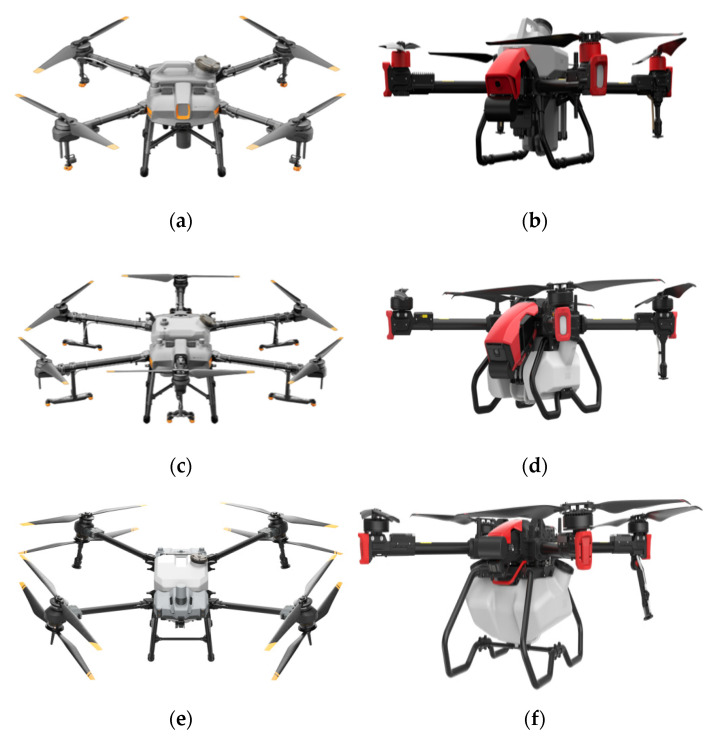
T series plant protection UAV and P series plant protection UAV. (**a**) DJI—T10. (**b**) XAG—P40. (**c**) DJI—T20. (**d**) XAG—P80. (**e**) DJI—T40. (**f**) XAG—P100.

Recently, the rapid development and widespread application of plant protection UAVs as one of the essential components in the Chinese agricultural plant protection industry have attracted much attention [1]. A plant protection UAV is an uncrewed aircraft consisting of a flight platform (fixed wing, helicopter, multiaxial aircraft), navigation flight control, and a three-part spraying mechanism used for plant protection operations in agriculture. UAVs can be operated by ground remote control or GPS flight control, and the spraying operators operate remotely to prevent exposure to pesticides, reduce spray drift and improve the safety of the spraying operations. Currently, the T Series plant protection UAV produced by Shenzhen DJI Innovation Technology Co., Ltd., and the P Series plant protection UAV produced by Guangzhou XAG Technology Co., Ltd., are the most commonly used in China; see Figure 1.

Compared with traditional methods of spraying and protecting agriculture and ground plant protection machinery, plant protection UAVs are more flexible, efficient, safe, affordable and adaptable. Furthermore, there is less risk that they will destroy cultivated land [2,3,4]. Plant protection UAVs do not need a runway for take-off and landing, which is especially suitable for areas with small fields and scattered fields and agricultural areas with dense dwellings [5]. Plant protection UAVs usually fly at a low altitude. The downwash airflow produced by the rotor increases droplet penetration to crops and there is good control. With the popularization and application of UAVs in pesticide spraying, the utilization rate of pesticides has dramatically increased. However, there are still problems such as pesticide waste and pollution caused by pesticide drift. The deposition effect of droplets on the target crops is an important index to evaluate the effect of farmland operation [6].

Currently, a field test is the primary method used to study the droplet deposition laws and effect of plant protection. Wang et al. [7] studied the influence of different operating heights on the spraying effect of a betel nut canopy by spraying allura red water stain with a quality score of 0.5% instead of a liquid pesticide. The results showed that the operating height had no significant effect on the droplet deposition at sampling points in each layer of the betel nut tree. Chen et al. [8], using a four-rotor plant protection UAV, confirmed the effect of the wind field under the multi-rotor UAV on the droplet deposition distribution by four spray trials. Qin et al. [9] studied the effect of operation height on the uniformity of droplets on plants, providing a technical basis for the optimized design, improved performance, and the correct use of spray devices. Kharim et al. [10] studied the optimal operating parameters during a six-rotor UAV spraying, performing field trials using different flight speeds (2, 4, and 6 m/s) at a constant height of 2 m above a rice canopy with an organic liquid fertilizer at different spraying speeds (0.75, 1.5, 2.25, and 3.00 L/min). Droplets were collected with water-sensitive paper as the sampler, and the deposition density was statistically analyzed. The test results showed that the best operating parameters to maximize droplet deposition density and uniformity were: flight height, 2 m; flight speed, 2 m/s; spray speed, 3.00 L/min. Guo et al. [11] conducted field tests to investigate the effects of flight parameters, droplet size, and crop phenotype on the droplet deposition density and deposition uniformity of a quad-rotor plant protection UAV. The test results show that the importance of the three factors is in the order of the flight parameters, crop phenotype, and droplet size. Wang et al. [12] performed pineapple spraying tests with plant protection UAVs at different meteorological conditions and flight heights. The results show that wind speed and droplet size are the main factors affecting the distribution and drift distance.

During the field experiment, it is necessary to enter the spraying region to arrange sampling points before the experiment. After the sampling points are collected, it is necessary to carry out the statistical calculation of the droplet deposition rate at the sampling points. The workload is quite large, and the evaluation results cannot be obtained immediately after the plant protection operation. It is challenging to obtain the droplet deposition distribution in the whole operation area according to the limited sampling points. Computational fluid dynamics (CFD) is a crucial simulation and analysis tool for studying the effect of plant protection. It plays an essential role in the field of plant protection. Simulation results have an important guiding significance for spraying decisions and influencing factor analysis in practical operations. The computational fluid dynamics can simulate the spray environment, consider the spray influence factors, predict the droplet deposition distribution and drift trend, and make predictions with high accuracy. It can effectively simulate the actual operation situation, which is conducive to establishing the droplet deposition and drift model [6].

Qi et al. [13] used CFD technology to establish the Hardi LB255 spray machine’s airflow field velocity and droplet deposition distribution models. Yang et al. [14] used the CFD method for an SLK-5 six-rotor plant protection UAV to establish a two-phase flow model under suspension spray conditions. The motion of different droplets in the suspended wind washing field indicates the droplet drift, motion, and deposition of multi-rotor plant protection UAVs. Sun et al. [15] investigated the effect of airflow on droplet drift under different spray conditions in 3D space based on the particle tracking techniques of a discrete phase model in CFD. The results showed that the distribution trend varies with wind speed and spray height; that is, the droplet deposition gradually decreases with an increase in spray height and wind speed. A prediction model for the droplet deposition rate was established based on this method. Duga et al. [16], considering the tree structure, canopy airflow, and sprayer motion, calculated orchard sprayer spray deposition and drift by establishing a 3D CFD model and experimentally validated it in apple orchards with different nozzle arrangements. Using numerical simulations and field tests, Zhang et al. [17] studied droplet migration in the non-target area of n-3 single rotor plant protection UAVs under particular flight and natural wind conditions. Hong et al. [18] developed an integrated CFD model to predict the wind speed distribution inside and around the canopy of an air-assisted sprayer, performing comparisons with the actual test results. The results show that the model is able to reasonably predict the air distribution of the air-assisted sprayer. Guo et al. [19] established a three-dimensional CFD model of a four-rotor plant protection UAV in hover and studied the distribution characteristics of the downwash wind field in hover; this laid a foundation for studying the motion law of spray droplets from the plant protection UAV. Wang et al. [20] established simulation regions based on CFD technology and set up initial conditions to study the deposition characteristics of droplets under multiple influencing factors. The results showed that as the crosswind velocity increased and the spray height decreased, the deposition area moved away from the nozzle. The spray is unsuitable when the crosswind speed is more significant than 3 m/s, or the spraying height is greater than 1.3 m. As the droplet size increases, the amount of deposition increases and the deposition area decreases. The change in spray angle has some influence on the distribution of droplet deposition and is the main factor affecting the deposition characteristics.

Using computational fluid dynamics to carry out numerical simulation can only calculate the droplet deposition distribution under a fixed set of parameters each time, which requires much calculation time. During the plant protection operation, the factors that affect the droplet deposition distribution, such as flight and environmental parameters, change continuously. Therefore, it is unrealistic to use computational fluid dynamics to obtain the droplet deposition distribution in real-time.

With the development of the Internet of Things technology, we can obtain real-time information about the flight position, height, flight speed and spraying speed through various UAV sensors. In real-time, we can obtain meteorological information such as temperature, humidity, wind speed and wind direction through a portable small weather station. In plant protection UAV operation, we can obtain the above information at a particular time interval. In the field of plant protection UAV, it is urgent to develop a model that can calculate the droplet deposition distribution in real-time according to the collected flight parameters and environmental information. In this case, we can accumulate the spray droplet deposition at each moment and get the deposition distribution of the spray droplets in the whole operating area. Lebeau et al. [21] developed a spray drift model called RTDrift to estimate the drift caused by ground sprayer machines. The sprayer was equipped with sensors measuring the operational parameters: spray pressure, boom height, and movement. Climatic parameters, including wind speed and direction, were measured using a 2D ultrasonic anemometer mounted on the sprayer. A diffusion–advection Gaussian tilted plume model at every successive boom position computed the spray drift deposits for each dropped class. The contribution of a single nozzle was calculated by integrating the individual puffs concerning time and the summation of the contributions of individual drop classes. The overall drift generated by the sprayer machine was obtained by adding the contributions of all the nozzles. The movement of the droplets ejected from the ground spraying machine is mainly affected by the environmental wind, so the Gaussian tilted plume model can be used to calculate the drift of the droplets and then obtain the deposition of the droplets on the ground.

Unlike the ground plant protection equipment, the droplets ejected by the plant protection UAV are farther away from the ground. The UAV spray still has problems, such as droplet drift and uneven spraying. The study of the laws governing the distribution of droplet deposition can enable calculation of the distribution of droplets in the operation area in a timely manner and objectively evaluate the quality of the operation after the plant protection operation. At the same time, the selection of job parameters can also be optimized according to the job quality requirements.

The distribution of droplet deposition is affected by various factors such as flight parameters, natural factors and spray equipment. In this study, we fixed the experimental UAV and spray equipment and ignored the effect of temperature and humidity on droplet evaporation. Flight height, speed, and crosswind speed were selected as the main influencing factors. This paper combines an SST k turbulence model and the semi-implicit method for pressure linked equations (SIMPLE) algorithm to simulate the downwash airflow field of six-rotor plant protection UAVs. The wind speed measurement experiment is conducted, and the comparison verifies the reliability of the numerical simulation. Liquid droplets are injected into the flow field to track the moving trajectory of a certain number of liquid droplets using the DPM model to obtain the deposition distribution of fog droplets in the ground target area. Using the inverse distance weighting (IDW) method based on the droplet deposition distribution in the 294 operation conditions, one can calculate the droplet deposition distribution when the three parameters change in a particular range. In the actual operation, by obtaining the flight height and speed of the plant protection UAV and the crosswind speed in real-time, we can calculate the deposition distribution of the droplets sprayed by the plant protection UAV in different locations at different times. The proposed method lays a foundation for the cumulative calculation of droplet deposition distribution during continuous operation of plant protection UAV. It provides a basis for objectively evaluating the operation quality of plant protection UAVs and optimizing the setting of operation parameters.

## 2. Materials and Methods

### 2.1. Plant Protection UAV

**Figure 2:**
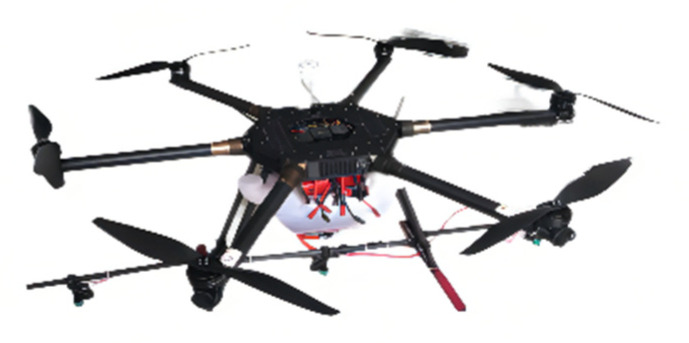
Harvest-1 plant protection UAV.

This paper reports on a study of a Harvest-1 plant protection UAV provided by Hebei Qiuze Intelligent Technology Co. Ltd., Baoding, China, depicted in Figure 2.

The main performance parameters of the UAV are shown in Table 1. Three VP110-015 fan-shaped nozzles were mounted directly 0.5 m below the left and right rotors and the fuselage. At 0.5 MPa operating pressure, the spray flow rate of a nozzle was 0.013 kg/s. The diameter median, D_50_, at 0.3 m below the nozzle was about 86.38 μm, whereas the mean diameter D_av_ was about 96.96 μm. The chosen test instrument was the Winner312 spray granularity analyzer produced by Jinan Weena Granticle Instrument Co., Ltd., Jinan, China. 

### 2.2. Physical Model

**Figure 3:**
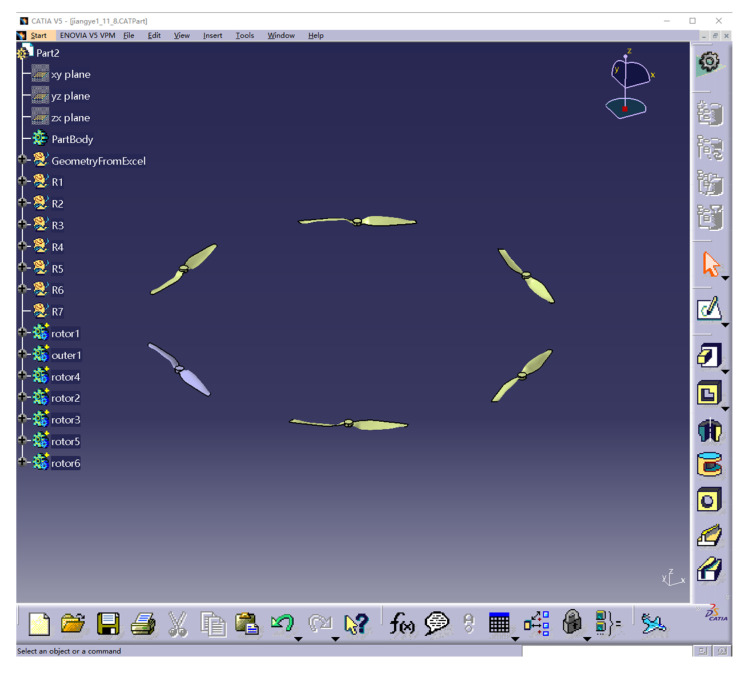
Physical model of six rotors for the Harvest-1 UAV.

The present trial adopted the method used in the literature [14]; the physical model was only generated for the six rotors, ignoring the body and other accessories. A physical model of the six rotors of the Harvest-1 plant protection UAV was generated using CATIA software, as shown in Figure 3. The rotor radius was 0.25 m, with an inter-axis distance of 0.685 m.

**Figure 4:**
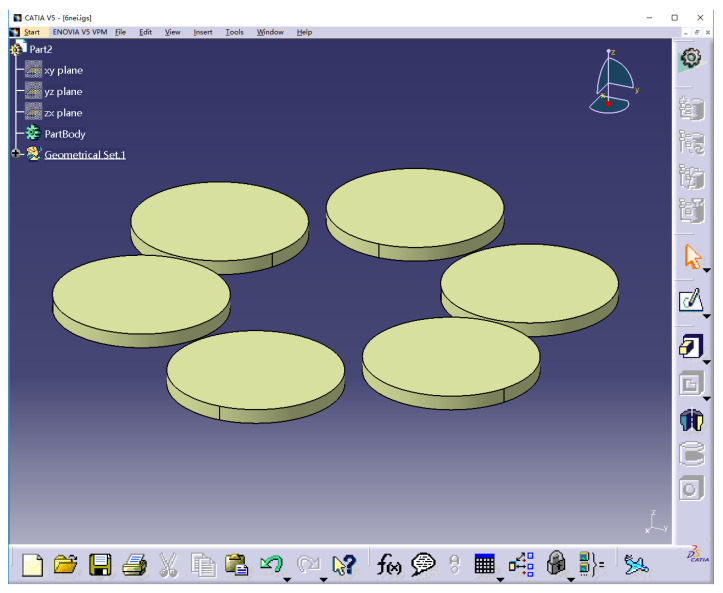
Physical model of moving fields.

Moving fields containing six rotors for six cylinders with a bottom radius of 0.3 m and height of 0.05 m, were generated using CATIA software, as shown in Figure 4.

**Figure 5:**
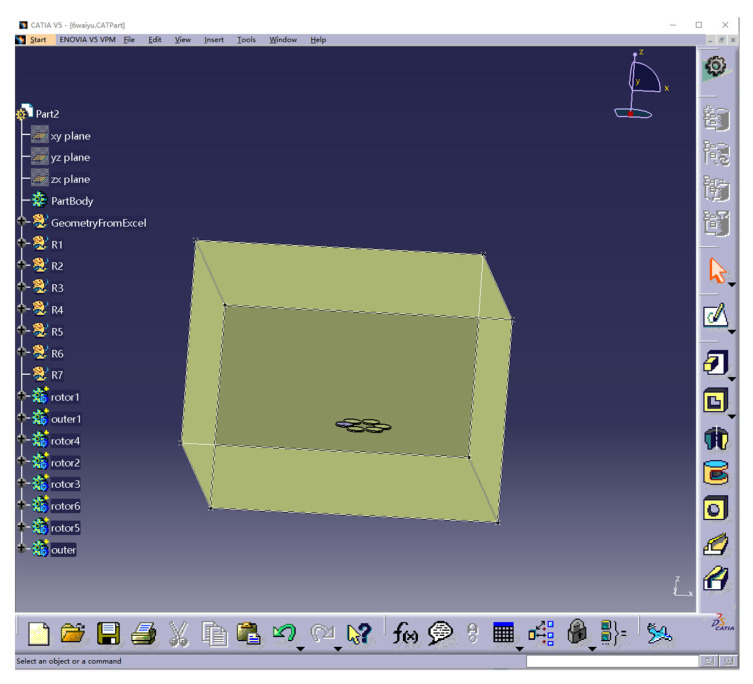
Physical model of external regions.

External regions containing the six rotors were generated using CATIA software. The outer region was a cube with a length and a width of 20 m, height of 7 m (because the UAV was 2 m above the ground), and six rotors located at the center of the cube and 2 m from the bottom region, as shown in Figure 5.

### 2.3. Grid Processing

**Figure 6:**
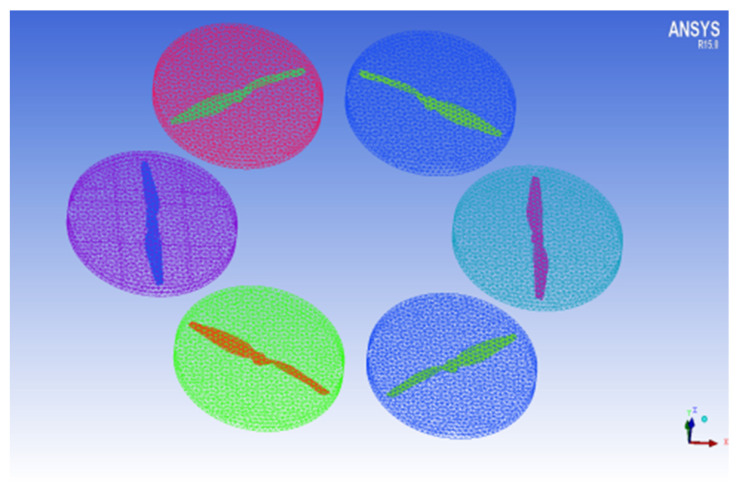
Grid division of the six rotor and the dynamic domain.

The generated physical model was imported into ICEM software for grid segmentation. Unstructured grids were used to divide the six rotors and dynamic domains, with approximately 5.08 million grids, as shown in Figure 6.

**Figure 7:**
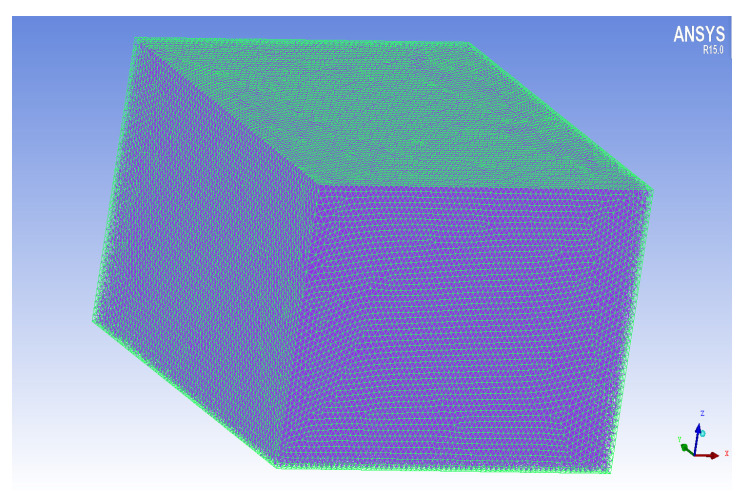
Grid division of external regions.

The external regions containing six rotors were divided, with about 1.6 million grids, as shown in Figure 7.

The dynamic domain and the static domain grids are fused, the lateral wind direction is in a positive direction of the *x*-axis, and the UAV flies in a negative *y*-axis direction.

### 2.4. Numerical Simulation Calculation of the Flow Field

**Figure 8:**
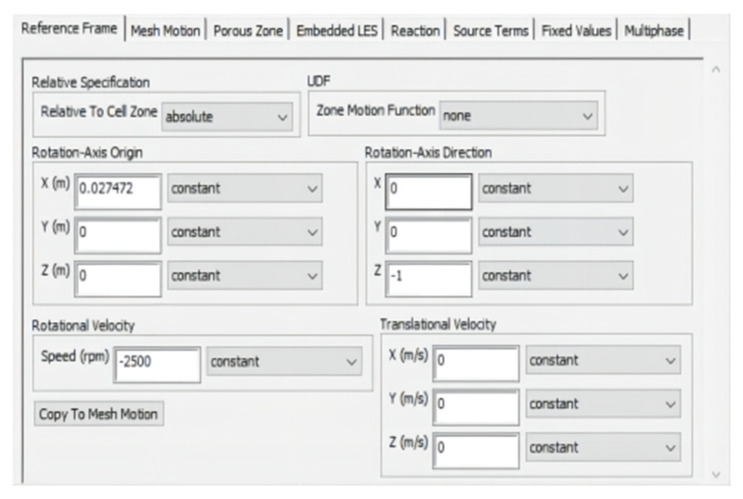
Settings of the cell zone conditions.

The divided grid file was opened in Fluent software, and the turbulence SST k-omega model was selected, whose mathematical general form is as follows:

(1) $$\begin{array}{l} \frac{\partial \left(\rho \psi \right)}{\partial t}+\frac{\partial \left(\rho \mu \psi \right)}{\partial x}+\frac{\partial \left(\rho \nu \psi \right)}{\partial y}+\frac{\partial \left(\rho w\psi \right)}{\partial z}=\\ \frac{\partial }{\partial x}\left(\zeta \frac{\partial \psi }{\partial x}\right)+\frac{\partial }{\partial y}\left(\zeta \frac{\partial \psi }{\partial y}\right)+\frac{\partial }{\partial z}\left(\zeta \frac{\partial \psi }{\partial z}\right)+S \end{array}$$

where *u*, *v*, and *w* are components of the velocity vector in the *x*, *y*, and *z* directions, *ψ* is the universal variable, *ζ* is the generalized diffusion coefficient, and *S* is the generalized source term. Frame Motion in the Cell Zone Conditions option was chosen. The rotation center of the six rotors was set, and the z-axis was set as the rotation axis. The rotor speed was 2500 rpm, which is the rotation speed in the hovering UAV and the two adjacent rotors had opposite directions of rotation, as shown in Figure 8.

**Figure 9:**
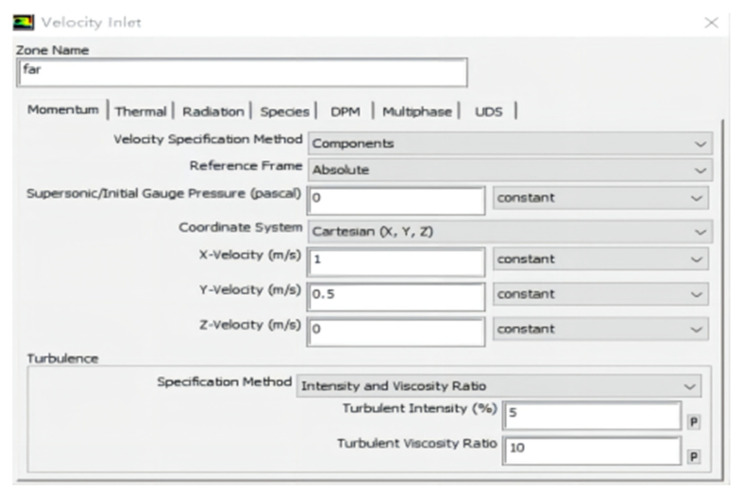
Settings of the boundary conditions.

The boundary conditions were set in the Boundary Conditions option, and the cylindrical surface containing the six rotors made up the interface. The plane around the outer was static, and the upper plane was the velocity-inlet. The front flight speed was 0, 0.5, 1, 1.5, 2, 2.5, and 3 m/s; the crosswind speed was set to values of 0, 0.5, 1, 1.5, 2, 2.5, and 3 m/s, as shown in Figure 9. The bottom plane, representing the ground, was set to be a wall.

After setting the boundary conditions, the SIMPLE algorithm was selected to calculate the flow field distribution. The convergence condition residual was set to 1 × 10^−5^ to calculate the convergence and obtain the flow field distribution of the whole region.

### 2.5. Combination of Experimental Parameters

Numerical simulations were performed in Fluent software using 294 combinations of experiment parameters. The flight heights were 2 m, 2.1 m, 2.3 m, 2.5 m, 2.7 m, and 3 m, the flight speeds were 0 m/s, 0.5 m/s, 1 m/s, 1.5 m/s, 2 m/s, 2.5 m/s, and 3 m/s, and the crosswind speeds were 0 m/s, 0.5 m/s, 1 m/s, 1.5 m/s, 2 m/s, 2.5 m/s, and 3 m/s, based on practical experience, as shown in Table 2, where Fh stands for flight height, Fs is flight speed, and Cs is crosswind speed.

## 3. Results and Analysis of Wind Speed Field

### Wind Speed Field Distribution under Different Combinations of Experiment Parameters

**Figure 10:**
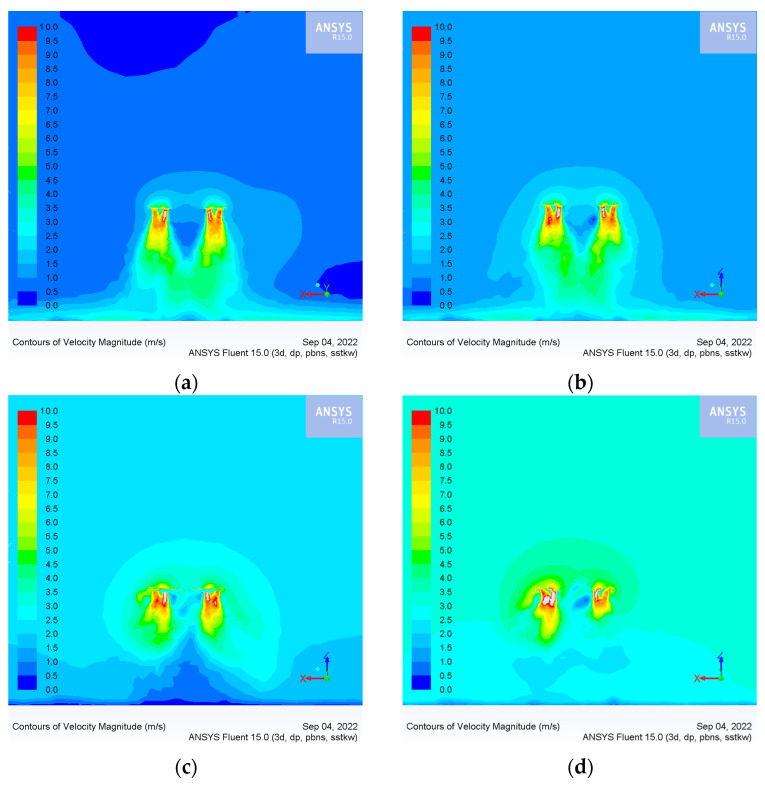
Wind speed field distribution of the xoz section with a flight height of 3 m, crosswind speed of 0 m/s, and different flight speeds: (**a**) UAV flight speed of 0.5 m/s; (**b**) UAV flight speed of 1 m/s; (**c**) UAV flight speed of 2 m/s; and (**d**) UAV flight speed of 3 m/s.

The distribution of the wind speed field is determined by four factors: flight height, flight speed, crosswind speed and rotor speed. The rotor speed is 2500 rpm, which is the rotation speed in the hovering UAV. In this experiment, when the UAV keeps hovering, the airflow opposite the flight direction is introduced to simulate the flight state of the UAV. The absolute value of the airflow speed is equal to the flight speed, and the direction is the opposite direction of the flight direction of the UAV. The *x*-axis is the flight direction, and the *y*-axis is the crosswind direction. Figure 10 shows the wind speed field distribution with a flight height of 3 m and crosswind speed of 0 m/s under different flight speed conditions.

As shown in Figure 10a,b, when the flight speed of the UAV does not exceed 1 m/s, the high-speed wind field is mainly distributed directly below the UAV rotor. At this time, the ejected droplets are applied down by the wind field, and most are deposited in the area directly below the UAV. As shown in Figure 10c,d, when flight and crosswind speed exceed 2 m/s, the relative airflow produces a vortex in the downwash wind field below the UAV and reduces the stability of the downwash wind field. At this time, droplets in the wind field move up and drift away from the UAV.

**Figure 11:**
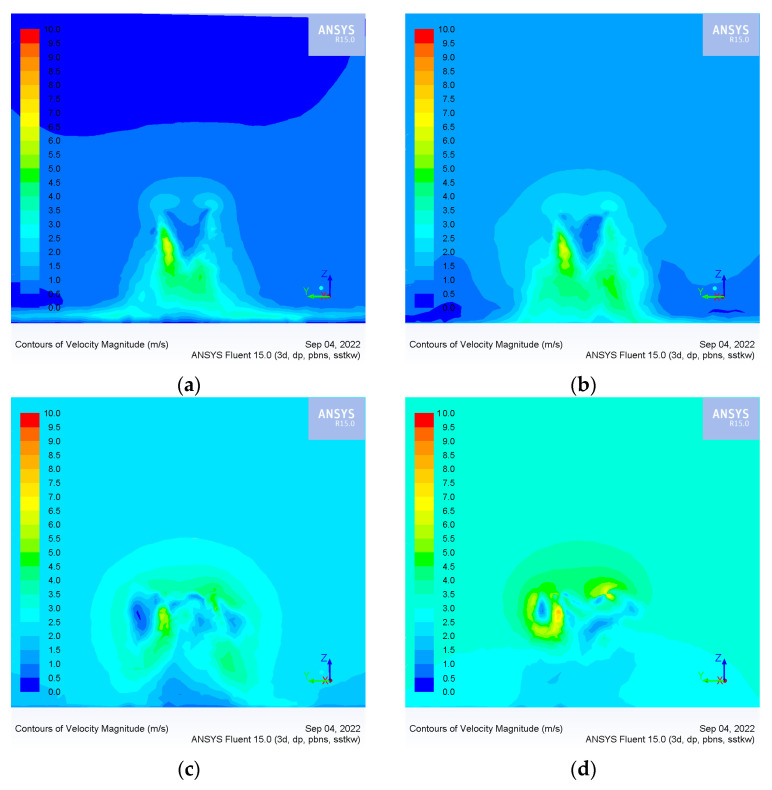
Wind speed field distribution of the yoz section with a flight height of 3 m, flight speed of 0 m/s, and different crosswind speeds: (**a**) crosswind speed of 0.5 m/s; (**b**) crosswind speed of 1 m/s; (**c**) crosswind speed of 2 m/s; (**d**) crosswind speed of 3 m/s.

Figure 11 shows the wind speed field distribution with a flight height of 3 m and flight speed of 0 m/s, with different crosswind speeds.

As shown in Figure 11a,b, when the crosswind speed does not exceed 1 m/s, the high-speed wind field is mainly distributed directly below the UAV rotor. At this time, the ejected droplets are applied down by the wind field, and most of them are deposited in the area directly below the UAV. Figure 11c,d show that when the crosswind speed exceeds 2 m/s, the high-speed wind fields are distributed above the UAV, and the wind field speed below gradually weakens. At this time, droplets in the wind field move upward and drift along the wind direction.

Through the numerical simulation of the wind speed field, we found that when the flight speed and the crosswind speed exceeds a specific range, the distribution of the wind speed field will change, which then affects the movement trajectory and deposition distribution of the droplets.

## 4. Experimental Verification of the Hovering Air Washing Field

**Figure 12:**
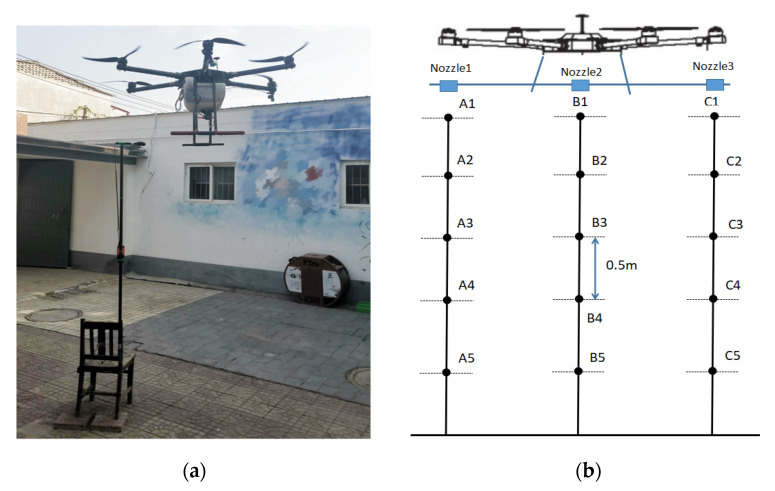
(**a**) wind speed test site; (**b**) measurement point layout.

To verify the accuracy of the numerical simulation of the UAV rotor wind field, a wind field test under a UAV hovering state was conducted. During the wind field test, the six-rotor plant protection UAV hovers at a ground heght of 3 m. The wind speed test point is placed below the left and right rotor and directly below the body, respectively, from the ground height of 0.5, 1, 1.5, 2, 2.5 m, a total of 15 test points. The “SW6036” type digital anemometer (produced by Guangzhou SWEVY Company, equipment accuracy ± 3%) is used to measure the speed in the Z direction of the 15 measuring points. The wind speed test site is shown in Figure 12a, the measurement point layout is shown in Figure 12b, the natural wind speed is 0.3~0.5 m/s, and the temperature is 26 °C.

The measured average value of the wind speed in the z direction of the 15 observation points and numerical simulated wind speed values at the corresponding point are shown in Table 3. The wind speed relative error is within 12%. The numerical calculation meets the accuracy requirements of the engineering calculation. It verifies the accuracy of the numerical model, which can be used to analyze the wind field of the six-rotor plant protection UAV.

## 5. Discrete Phase Model

After the wind field calculation converged, the discrete phase of droplets was injected. Three flat-fan atomization nozzles were set directly below the left and right rotors and the body’s center. At 0.5 MPa operating pressure, the spray flow rate was 0.013 kg/s, with a spray half angle of 55°, an orifice width of 1.5 × 10^−5^ m, and a diffusion angle of 6°. The above data were measured in the laboratory. The chosen test instrument was the Winner 312 spray granularity analyzer produced by Jinan Weena Granticle Instrument Co. Ltd.

Setting the boundary type of the upper and surrounding planes of the outer domain to “escape”, the lower plane boundary type representing the ground was set as “trap”. The moving trajectories of the droplets in the wind field and the position of the droplets at different times were calculated using the discrete phase model.

### 5.1. Statistical Calculation of the Droplet Deposition Rate

The plane right-angle coordinate system was established with the position directly below the UAV as the origin, and the plane area of 20 m × 20 m was divided into grids of 0.5 m × 0.5 m, with a total of 1600 small regions.

The droplet deposition rate within the *j* region was computed using the following formula:

(2) $$\begin{array}{l} C_{j}=\frac{\sum_{i=1}^{3}n_{j}^{\left(i\right)}}{N}\times 100\%\;(j=1,2,\ldots 1600)\;\\ n_{j}^{\left(i\right)}\left(i=1,2,3;j=1,\ldots 1600\right) \end{array}$$

where $n_{j}^{\left(i\right)}$ is the number of droplets deposited in the *j* region which were ejected from the *i* nozzle, and N is the total number of droplets ejected from the three nozzles.

### 5.2. Determination of the Number of Particle Streams

When simulating droplet motion using a discrete phase model, the number of particle streams ejected by the nozzle must be specified, because this determines our calculation accuracy and speed. We conducted comparative tests under operating conditions of a flight height of 3 m, flight speed of 0 m/s, and crosswind speed of 0 m/s, setting the number of particle streams ejected by a single nozzle to 3000, 5000, and 7000. The number of grids that captured the particles, and the maximum and minimum deposition rates and deposition recovery rate in the grid were calculated. The deposition recovery rate refers to the proportion of the number of droplets captured on the ground in relation to the number of droplets sprayed. The comparative results are shown in Table 4.

**Figure 13:**
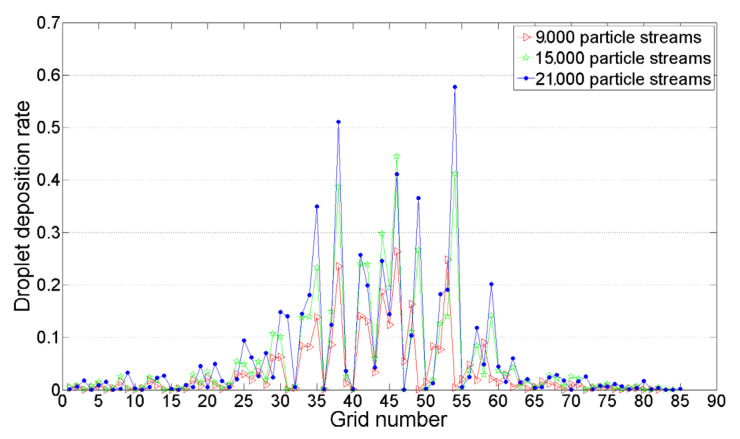
Droplet deposition rate within the same grid with different number of particle streams.

The droplet deposition rates within the same position grid were compared, and the results are shown in Figure 13.

The statistical results show that there was little difference in the droplet deposition rate within the same grid with a different number of particle streams; thus, the number of particle streams had little impact on the droplet deposition distribution. Considering both calculation accuracy and speed, we determined the number of particle streams for a single nozzle employed in the experiment to be 3000.

After the number of tracking particle streams was determined, 9000 particles were injected into the wind field under 294 operating conditions to obtain the deposition position of droplets in different flight states.

### 5.3. Effect of Flight Speed on the Distribution of Droplet Deposition

**Figure 14:**
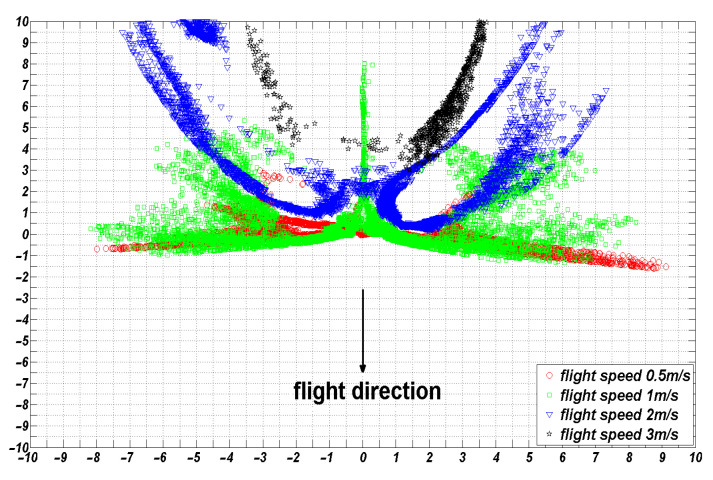
Droplet deposition situation at a flight height of 3 m, crosswind speed of 0 m/s, and different flight speeds.

With fixed a flight height and crosswind speed, different flight speeds were selected for the numerical simulations to obtain the deposition distribution of droplets on the ground, as shown in Figure 14.

**Figure 15:**
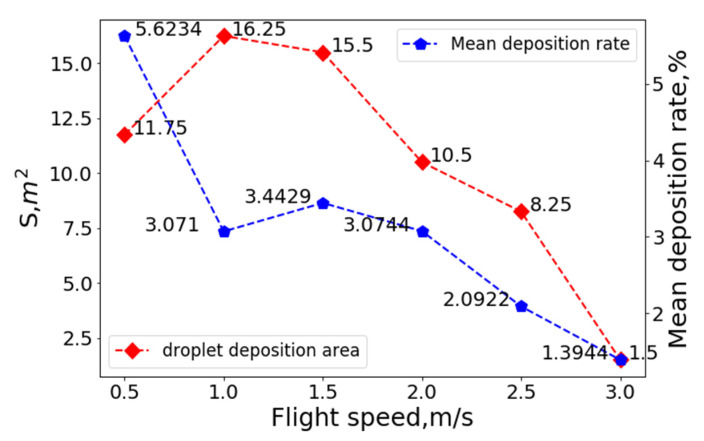
The variation curves of the coverage area, S, and mean droplet deposition rate at a flight height of 3 m, crosswind speed of 0 m/s, and different flight speeds.

Regions with a droplet deposition rate greater than 1% were considered practical deposition regions. The number of deposition regions under different combinations of operating parameters multiplied by the area of each small region was the droplet deposition coverage area, S. The variation curves of the coverage area, S, and mean droplet deposition rates at a flight height of 3 m, crosswind speed of 0 m/s, and different flight speeds are shown in Figure 15:

As shown in Figure 13, in the absence of crosswind, when the flight speed was less than 1 m/s, the downwash wind field under the rotor affected the droplets, and the droplet deposition distribution was mainly concentrated in the area below the UAV. With an increased flight speed, the ejected droplets gradually eliminated the influence of the downwash wind field under the rotor and drifted toward the area behind the UAV. When the flight speed reached 3 m/s, most droplets were emitted from the observation area and drifted away. As shown in Figure 14, the coverage area was only 1.5 m^2^, which is unsuitable for spraying operations.

As detailed in Figure 15, at a flight height of 3 m, the droplet coverage area and the mean value of the droplet deposition distribution gradually decreased with the increasing flight speed. The effective coverage area of the droplet deposition was maximized at a flight speed of 1 m/s. With the increase in flight speed, the effective coverage area became increasingly smaller. The mean value of the droplet deposition distribution in each small unit was also gradually reduced, and the droplet drift became increasingly severe. Flight speed is one of the critical factors affecting the distribution of droplet deposition.

### 5.4. Effect of Crosswind Speed on the Distribution of Droplet Deposition

**Figure 16:**
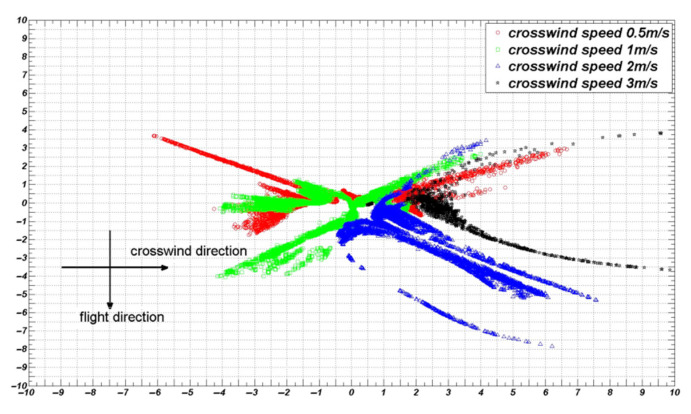
Droplet deposition situation at a flight height of 3 m, flight speed of 0 m/s, and different crosswind speeds.

At a fixed flight height and flight speed, different crosswind speeds were selected for numerical simulations to obtain the deposition distribution of droplets on the ground, as shown in Figure 16.

**Figure 17:**
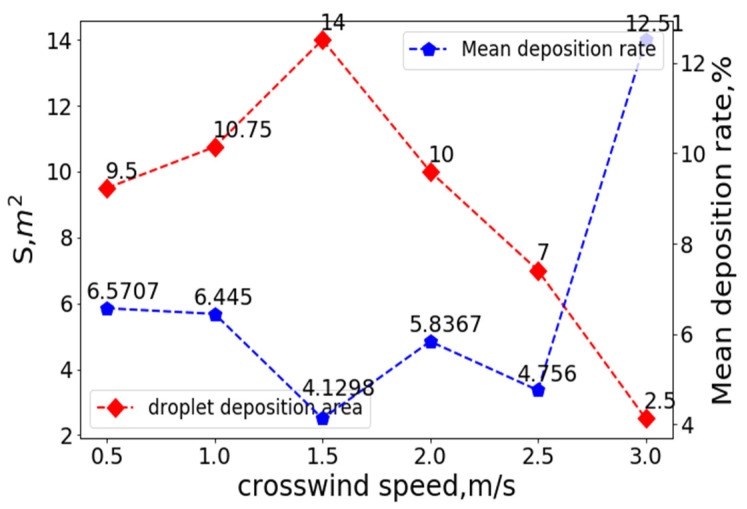
Variation curves of the coverage area, S, and mean droplet deposition rate at a flight height of 3 m, flight speed of 0 m/s, and different crosswind speeds.

The variation curves of the coverage area, S, and mean droplet deposition rate at a flight height of 3 m, flight speed of 0 m/s, and different crosswind speeds are shown in Figure 17.

As depicted in Figure 16, at a flight height of 3 m, when the crosswind speed was less than 1 m/s, the movement of the droplets was mainly affected by the downwash wind field produced by the UAV rotors, and the droplets were distributed on both sides of the UAV. When the crosswind speed was more significant than 1 m/s, the droplets’ movement was mainly affected by the crosswind, drifting towards the direction of the crosswind. The deposition distribution of the droplets on the ground was mainly concentrated on the right side of the UAV.

As shown in Figure 17, at a flight height of 3 m and when the crosswind speed was 1.5 m/s, the effective coverage area of the droplet deposition was maximized and the mean value of droplet deposition distribution in each small unit was minimized. With the increase in the crosswind speed, the effective coverage area became increasingly smaller, and the mean value of the droplet deposition distribution in each small unit became increasingly larger. The droplet distribution was gradually concentrated towards the direction of the crosswind. The crosswind speed is one of the key factors affecting the distribution of droplet deposition.

### 5.5. Effect of Flight Height on the Distribution of Droplet Deposition

**Figure 18:**
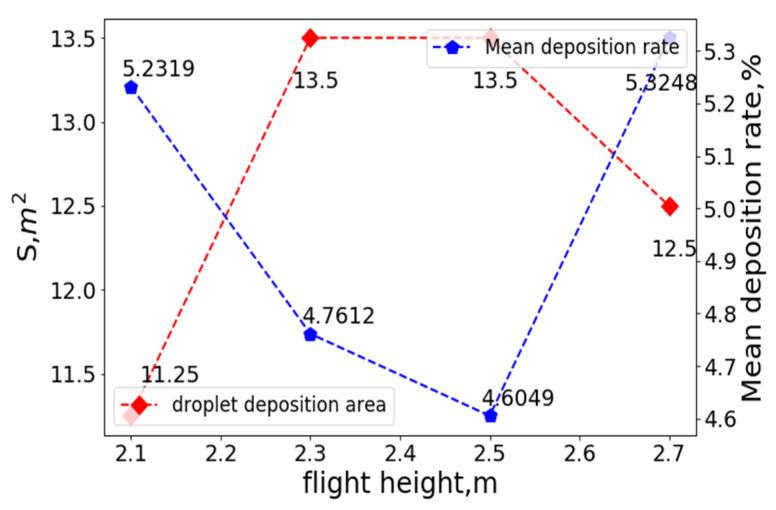
The variation curves of the coverage area, S, and mean droplet deposition rate at a crosswind speed of 0 m/s, flight speed of 0 m/s, and different flight heights.

At a fixed flight and crosswind speed, different flight heights were selected for numerical simulations to determine the deposition distribution of droplets on the ground. The variation curves of the coverage area, S, mean droplet deposition rate at a crosswind speed of 0 m/s, flight speed of 0 m/s, and different flight heights are shown in Figure 18.

As depicted in Figure 17, when the flight height was 2.1 m, the effective coverage area of the droplet deposition was minimized, and the mean value of droplet deposition distribution in each small unit was maximized. Under these conditions, due to the low flight height of the UAV, the downward airflow caused the droplets to be quickly deposited in a smaller range below the UAV, and the mean droplet deposition rate in each grid within the deposition area was very high. When the flight height was 2.5 m, the effective coverage area of the droplet deposition was maximized, whereas the mean value of the droplet deposition distribution in each small unit was minimized. With the increase in UAV flight height, the downward speed of the airflow field near the ground gradually weakened, the airflow rolled upward near the ground, and some of the droplets’ diffusion underneath the rolling airflow expanded the deposition area; at the same time, the average deposition rate of the droplets in each grid decreased.

In addition to these three influencing factors, regarding the UAV itself, the structure, rotor number, relative position, nozzle type, nozzle number, nozzle location, and spray pressure are the key factors affecting the distribution of droplet deposition. Before applying the droplet deposition distribution model to the actual operation, we fixed the influencing factors of the UAV’s structure. We selected flight height, flight speed, and crosswind speed as inputs for the model.

The deposition distribution of droplets under a combination of the above three factors showed great randomness. With the continuous changes in the three operation parameters, the deposition of droplets on the ground exhibited a continuous trend. Based on the droplet deposition situation under the combination of 294 operating parameters, the deposition distribution situation of the droplets within a certain range can be calculated by interpolation.

## 6. Droplet Deposition Distribution Prediction Method based on IDW

Inverse distance weighting (IDW) is a standard method for spatial interpolation: the basic idea is to define the interpolation function as the weighted average of the function values of each data point. In conducting the interpolation, the influence of observation points on the internal interpolation points decreases with the increasing distance between them.

We introduced vector *P* = [*h,v,w*], which represents a combined vector of a job parameter, where *h* indicates the flight height (m), *v* indicates the flight speed (m/s), and *w* indicates the crosswind speed (m/s). The data were first normalized with the following normalization formula:

(3) $$x^{*}=\frac{x-x_{\min}}{x_{\max}-x_{\min}}\;$$

After the normalization, the operation parameter vector was recorded as $P^{*}=[h^{*},v^{*},w^{*}]$.

The deposition distribution rates within the 1600 grids under each job parameter obtained from the experiment were deposited in a matrix:

$$X=\left(\begin{array}{ccccc} x_{1}^{\left(1\right)} & x_{1}^{\left(2\right)} & x_{1}^{\left(3\right)} & \vdots & x_{1}^{\left(294\right)}\\ x_{2}^{\left(1\right)} & x_{2}^{\left(2\right)} & x_{2}^{\left(3\right)} & \vdots & x_{2}^{\left(294\right)}\\ x_{3}^{\left(1\right)} & x_{3}^{\left(2\right)} & x_{3}^{\left(3\right)} & \vdots & x_{3}^{\left(294\right)}\\ \vdots & \vdots & \vdots & \vdots & \vdots \\ x_{1600}^{\left(1\right)} & x_{1600}^{\left(2\right)} & x_{1600}^{\left(3\right)} & \vdots & x_{1600}^{\left(294\right)} \end{array}\right)$$

where $x_{j}^{\left(i\right)}\left(i=1,2,\ldots 294,j=1,2,\ldots 1600\right)$ represents the droplet deposition rate within the *i*th grid under the *j*th operation parameter combination. The introduced vector $Y={\left(y_{1},y_{2},y_{3}\ldots y_{1600}\right)}^{T}$ represents the droplet deposition rate within 1600 grids under a combination of job parameters to be predicted. Y is calculated as follows:

(4) $$Y=\sum_{j=1}^{n}\omega_{j}X_{j}$$

(5) $$\omega_{j}=\frac{\frac{\text{1}}{\text{dist(}P^{\text{*}},P_{j}^{\text{*}}{)}^{u}+\varepsilon }}{\sum_{j=1}^{n}\frac{1}{\mathrm{dist(}P^{*},P_{j}^{\text{*}}{)}^{u}+\varepsilon }},\sum \omega_{j}=1$$

where $\omega_{j}$ represents the weight of the deposition rate under the *j*th parameter combination, where

(6) $$\mathrm{dist}(P^{*},P_{j}^{*})=\sqrt{{\left(h^{*}-h_{j}^{*}\right)}^{2}+{\left(v^{*}-v_{j}^{*}\right)}^{2}+{\left(w^{*}-w_{j}^{*}\right)}^{2}}$$

represents the distance between the *j*th operation parameter combination and the combination of operating parameters to predict the deposition rate, and *u* is the power value of the distance. *ε* = 1 × 10^−6^ is taken to avoid the denominator equaling zero.

To determine the best power value, *u*, in the model, the sample data were divided into training data (70%) and test data (30%), and the model was determined with the training weight, with the model’s accuracy tested using the test data. The indicators used to evaluate accuracy were mean absolute error (MAE) and root mean square error (RMSE):

(7) $$\begin{array}{l} MAE=\frac{1}{m}\sum_{i=1}^{m}\left|Y_{io}-Y_{it}\right| \end{array}$$

(8) $$RMSE=\sqrt{\frac{1}{m}\sum_{i=1}^{m}{\left(Y_{io}-Y_{it}\right)}^{2}}\;$$

where $Y_{\mathrm{io}}$ and $Y_{\mathrm{it}}$ are the predicted and measured values of the *i*th test samples, respectively. The distance power, *u*, was 1–10 for the comparison test. The results are shown in Table 5, minimizing both the MAE and RMSE at *u* = 4. Thus, *u* = 4 was determined to be the model parameter value.

### Some Cases of Numerical Simulations

Case 1: Flight height of 3 m, flight speed of 2 m/s, flight direction in the *y*-axis-positive half-axis direction, crosswind speed of 2 m/s, and crosswind direction in the *x*-axis-positive half-axis direction.

**Figure 19:**
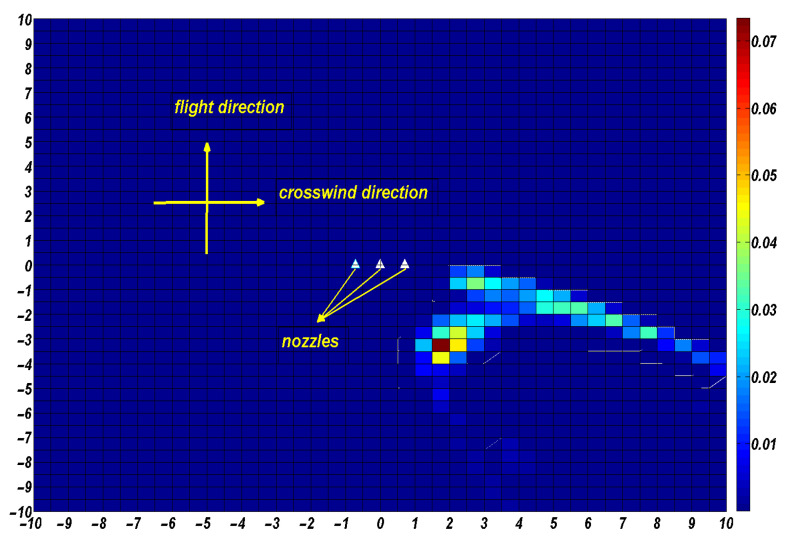
Droplet deposition distribution at a flight height of 3 m, crosswind speed of 2 m/s, and flight speed of 2 m/s,flight direction in the *y*-axis-positive half-axis direction.

At this point, the UAV was flying in the opposite direction to that of our simulation experiment. We calculate the deposition position of the droplets on the ground, using the current flight height, flight speed, and crosswind speed as inputs, and then find the symmetry points of these points about the *x*-axis. The position of all symmetry points is the deposition position of the droplet in its current state. Finally, the deposition distribution on the ground is obtained through statistical calculation, as shown in Figure 19.

Case 2: Flight height of 3 m, flight speed of 2 m/s, flight direction in the *y*-axis-negative half-axis direction, crosswind speed of 2 m/s, and the crosswind direction in the *x*-axis positive half-axis direction.

**Figure 20:**
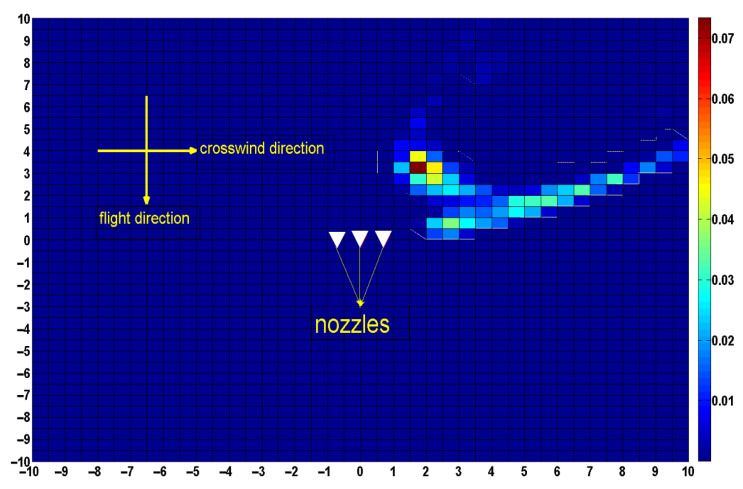
Droplet deposition distribution at a flight height of 3 m, crosswind speed of 2 m/s, and flight speed of 2 m/s,flight direction in the *y*-axis-negative half-axis direction.

At this time, the flight direction of the UAV is opposite to that in case 1; the other parameters took the same values as in case 1. The droplet deposition position in Case 2 and Case 1 is symmetrical about the *x*-axis, as shown in Figure 20.

Case 3: Flight height of 3 m, the flight speed of 1 m/s, flight direction in the *y*-axis-negative half-axis direction, crosswind speed of 2 m/s, flight direction in the *x*-axis-positive half-axis direction, and flight direction clip angle of 45°.

**Figure 21:**
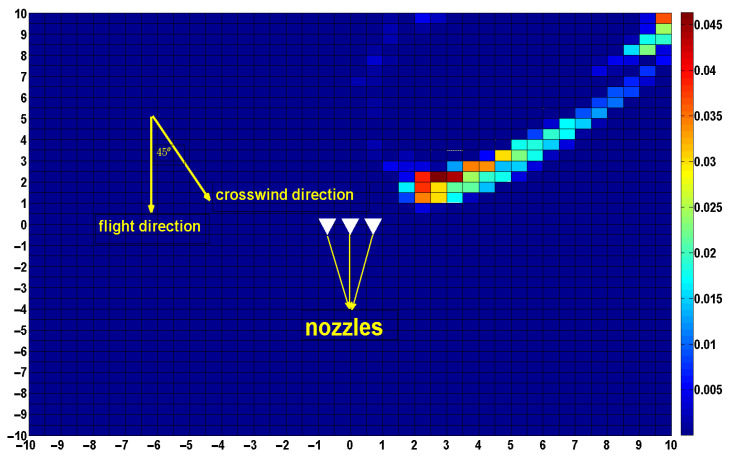
Droplet deposition distribution at a flight height of 3 m, crosswind speed of 2 m/s, crosswind and flight angle of 45°, and a flight speed of 1 m/s.

At this point, the wind direction is not perpendicular to the flight direction of the UAV, and it needs to be decomposed along the *x*-axis and *y*-axis. After the decomposition, the actual flight speed is 1 + 2 × cos45°, and the crosswind speed is 2 × cos45°. The results of droplet deposition calculated by substituting the above data as input are shown in Figure 21.

## 7. Conclusions

This paper studied the droplet deposition distribution of a six-rotor plant protection UAV provided by Hebei Qiuze Intelligent Technology Co., Ltd. using CFD numerical simulation, and drew the following conclusions:

1. In the actual plant protection operation, the UAV’s structure, the nozzle’s position and type, the spray flow rate and other factors need to be fixed in advance. The main factors affecting the distribution of droplet deposition are flight height, flight speed, crosswind speed. We find that when flight and crosswind speed do not exceed 1 m/s, the high-speed wind field is mainly distributed directly below the UAV rotor. At this time, the ejected droplets are applied down by the wind field and most of them are deposited in the area directly below the UAV. When flight and crosswind speed exceed 2 m/s, the relative airflow produces a vortex in the downwash wind field below the UAV and reduces the stability of the downwash wind field. At this time, droplets in the wind field move up and drift away from the UAV.
2. After obtaining the wind speed field distribution under the combination of 294 operating parameters, the discrete droplet phase is injected, and the trajectory of the movement of a certain number of particle streams in the wind speed field at different times is tracked using the discrete phase model. The comparative test determined the number of tracked particle streams as 9000 in terms of calculation accuracy and speed.
3. By calculating the droplet deposition distribution under 294 combinations of operating parameters, we found that the droplet deposition distribution exhibited a significant degree of randomness, and it is not easy to build models using data-fitting methods. Based on the distribution of droplet deposition under the combination of operating parameters in 294 sites, we can calculate the distribution of droplet deposition using an interpolation method.
4. We chose the inverse distance weight (IDW) interpolation method to calculate the distribution of droplet deposition The corresponding treatment scheme is given for some special cases. The feasibility of the method is illustrated through three specific computational cases. This method can calculate the deposition distribution of the droplets sprayed at the position on the ground based on the operation parameters collected during the operation of the plant protection UAV at a particular time.
5. Our proposed method is to calculate the deposition distribution of the droplets at a certain moment. In the field experiments, the continuous spraying method was used for the operation. An investigation of the methods to accumulate the droplet deposition distribution obtained at different times will be undertaken later. This portion of the field experiment will be supplemented in subsequent studies. In this experiment, most of the droplets drifted out of our study area when flight and crosswind speed exceeded 3 m/s. This limits the applicability of the method to some extent. In subsequent research, we will expand the external regions of the grid during the simulation and more comprehensively grasp the rules governing the movement of droplets.

## Figures and Tables

**Table 1:** Main parameters of the Harvest-1 plant protection UAV.

Parameter	Value
Flight Speed (m/s)	≤7
Maximum Wheelbase (mm)	1370
Load Weight (kg)	30
Rotor Radius (m)	0.25
Nozzle Type	VP110-015
Number of Nozzles	3
Spray Pressure (MPa)	0.5
Rate of Flow (kg/s)	0.013
Spray Half Angle (°)	55

**Table 2:** 294 combinations of experiment parameters.

Test Order	Fh (m)	Fs (m/s)	Cs (m/s)	Test Order	Fh (m)	Fs (m/s)	Cs (m/s)
1	2	0	0	…	…	…	…
2	2	0	0.5	292	3	3	2
3	2	0	1	293	3	3	2.5
…	…	…	…	294	3	3	3

**Table 3:** Experimental and simulated values of z-direction velocity of measurement points.

Measurement Point Serial Number	Experimental Value (m/s)	Simulated Value (m/s)	Relative Error (%)
A1	9.41	9.13	2.98
A2	7.82	7.32	6.39
A3	6.01	5.81	3.33
A4	4.51	4.25	5.76
A5	4.12	3.82	7.28
B1	1.51	1.42	5.96
B2	4.76	4.32	9.24
B3	4.02	4.24	5.47
B4	3.36	3.18	5.36
B5	4.02	3.63	9.70
C1	9.01	8.47	5.99
C2	8.02	7.86	2.00
C3	5.10	4.49	11.96
C4	4.50	4.10	8.89
C5	4.00	3.78	5.50

**Table 4:** Droplet deposition at different number of particle streams.

Number of Particle Streams	The Number of Grids Captured the Particles	MaximumDeposition Rate	Minimum Deposition Rate	Deposition Recovery Rate
3 × 3000	83	8.8%	0.001%	99.13%
3 × 5000	84	8.9%	0.001%	99.24%
3 × 7000	85	8.3%	0.001%	98.84%

**Table 5:** MAE and RMSE for different power values.

Power Value, *u*	MAE	RMSE
**1**	0.003100	0.002259
**2**	0.002892	0.002063
**3**	0.002710	0.001912
**4**	0.002669	0.001899
**5**	0.002718	0.001957
**6**	0.002781	0.002023
**7**	0.002833	0.002074
**8**	0.002870	0.002109
**9**	0.002896	0.002132
**10**	0.002913	0.002147

## Data Availability

The data presented in this study are available on request from the corresponding author.
